# Age Discrimination During the COVID-19 Pandemic: Associations With Daily Well-Being

**DOI:** 10.1093/geroni/igab046.682

**Published:** 2021-12-17

**Authors:** Lydia Ong, Patrick Klaiber, Anita DeLongis, Nancy Sin

**Affiliations:** University of British Columbia, Vancouver, British Columbia, Canada

## Abstract

During the COVID-19 pandemic, ageist attitudes have been pervasive in public discourse, interpersonal relationships, and medical decision-making. For example, older adults have been portrayed as vulnerable while younger adults have been portrayed as reckless. The current study examined age discrimination during COVID-19 and associations with daily affect and physical symptoms. Positive events and age were examined as moderators. From March to August 2020, 1493 participants aged 18-91 (mean=40) in the U.S. and Canada completed surveys for seven consecutive evenings about discrimination, positive events, affect, and physical symptoms. Multilevel models controlled for age, race, income, education, sample (university students vs. community), and country of residence. Results indicated that individuals who reported more age discrimination had higher negative affect (b=36.44, SE=3.97), lower positive affect (b=-19.07, SE=4.10), and increased physical symptoms (b=3.85, SE=0.49; p<0.001 for all), compared to those with fewer reports of age discrimination. Within-persons, days with age discrimination were associated with higher negative affect (b=3.66, SE=1.36, p=0.008), lower positive affect (b=-2.60, SE=1.23, p=0.037), and increased physical symptoms (b=0.26, SE=0.11, p=0.02), compared to days on which age discrimination was not reported. Positive events moderated the between-person association of age discrimination with physical symptoms such that individuals with more age discrimination and more frequent positive events reported fewer daily physical symptoms than those with more age discrimination and less frequent positive events. Age did not moderate the associations. Age discrimination was associated with poorer daily well-being during the COVID-19 pandemic and may have long-term impacts on intergenerational solidarity and attitudes toward aging.

